# Fucoidan-Functionalized Nanoparticles for Antimycobacterial Therapy: A Promising Approach against Drug-Resistant *Mycobacterium tuberculosis* Strains

**DOI:** 10.1021/acsomega.6c01348

**Published:** 2026-07-03

**Authors:** Luanna de Ângelis Correia de Sousa, José António Couto Teixeira, Ana Lucia Figueiredo Porto, Jaqueline Rodrigues da Silva, Mariane Cajubá de Britto Lira Nogueira, Fábio Rocha Formiga, Maria Carolina Accioly Brelaz de Castro, Yuri José de Albuquerque Silva, Romario Martins Araujo, Danielle Martiniano da Silva Rodrigues, João Paulo de Lucena Laet, José Luiz de Oliveira Magalhães, Giovanna Gabriela Pedroza Rodrigues, Lílian Maria Lapa Montenegro

**Affiliations:** † Department of Immunology, Aggeu Magalhães Institute, 92923 Oswaldo Cruz Foundation, 50740-600 Recife, Pernambuco, Brasil; ‡ Department of Biological Engineering, University of Minho, 4710−057 Braga, Portugal; § Academic Center of Vitória, Federal University of Pernambuco, 55608-680 Vitória de Santo Antão, Pernambuco, Brasil; ∥ Laboratory of Imunophathology Keizo-Asami, Federal University of Pernambuco, 50670-901 Recife, Pernambuco, Brasil; ⊥ Department of Animal Morphology and Physiology, Federal Rural University of Pernambuco, 52171-900 Recife, Pernambuco, Brasil; # Center for the Social Sciences of Nature, Federal University of Pernambuco, 50740-560 Recife, Pernambuco, Brasil; ∇ Biosafety Level 3 Laboratory, Aggeu Magalhães Institute, Oswaldo Cruz Foundation, 50740-600 Recife, Pernambuco, Brasil

## Abstract

Tuberculosis remains one of the leading causes of death from infectious diseases. Poly­(isobutyl cyanoacrylate) (PIBCA) polymeric nanoparticles coated with fucoidan were developed as a targeted drug delivery system for rifampicin (RIF) against *Mycobacterium tuberculosis* (Mtb), including multidrug-resistant strains. The nanoparticles were prepared by anionic emulsion polymerization and characterized for size, surface charge, morphology, stability, and encapsulation efficiency. The formulation exhibited a mean diameter of 400–480 nm, a negative zeta potential (−47.9 ± 1.27 mV), spherical morphology, and colloidal stability for over 30 days, with encapsulation efficiency between 40–50%. In vitro assays demonstrated antimycobacterial activity, with MIC values of 0.412 μg/mL for drug-sensitive strains and 1.238 μg/mL for drug-resistant strains. Synergistic interactions were observed when combined with standard anti-tuberculosis drugs. The nanoparticles showed low cytotoxicity (80–96% cell viability) and concentration- and time-dependent intracellular bactericidal activity in infected macrophages. Cellular uptake studies confirmed efficient internalization, consistent with fucoidan-mediated targeting of macrophage scavenger receptors. These findings highlight fucoidan-coated PIBCA nanoparticles as a promising nanotechnological strategy to enhance rifampicin delivery and efficacy against tuberculosis, particularly in drug-resistant infections.

## Graphical abstract

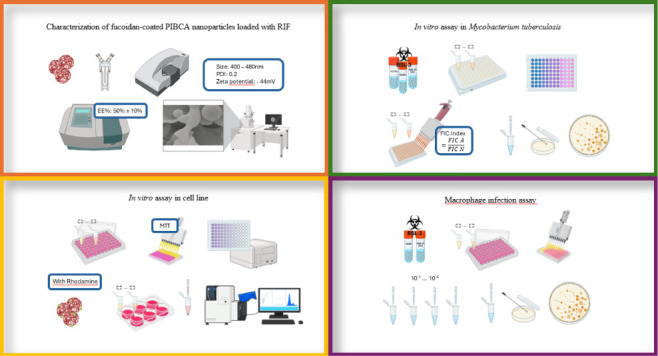

## Introduction

Tuberculosis (TB) is a chronic infectious disease caused by *Mycobacterium tuberculosis* (Mtb), an acid-fast bacillus and facultative intracellular pathogen that typically affects the lungs but may also infect other organs of the body, resulting in the extrapulmonary form of the disease.^1,2^ The World Health Organization (WHO) recognizes TB as the leading cause of death from a single infectious agent worldwide. Each year, the WHO reports approximately 10 million new cases and 1.5 million TB-related deaths.^3^

TB is a curable disease, treated with a standard regimen of four orally administered antibiotics over a six-month period.^4^ Rifampicin (R) is one of the first-line antibiotics used in combination with isoniazid (H), pyrazinamide (Z), and ethambutol (E) for 2 months, followed by RH for an additional 4 months. In drug-sensitive *Mycobacterium tuberculosis* strains, rifampicin (RIF) inhibits the activity of DNA-dependent RNA polymerase by forming a stable complex with the enzyme, thereby suppressing RNA synthesis initiation. Along with isoniazid, it is one of the drugs with the highest sterilizing capacity.^5,6^ However, the incidence of resistance to these drugs is steadily increasing. Rifampicin-resistant or multidrug-resistant tuberculosis (MDR-TB, resistant to both RIF and isoniazid), referred to as MDR/RR-TB, accounted for 400.000 new cases in 2023 according to WHO data, with a therapeutic success rate of around 68%, compared with cure rates above 88% for conventional treatments.^3^

Mycobacterial drug resistance involves the interaction of several factors, including treatment duration, the bacillus’ mutational and adaptive capacity, as well as poor patient adherence to therapy, mainly due to drug-related adverse effects.^7^ RIF, for example, may cause adverse effects such as headache, fatigue, mental confusion, and even hepatotoxicity. The severity and frequency of these complications are associated with drug dose and duration of treatment, which typically lasts throughout the entire therapeutic course. Moreover, the oral route of administration and the drug’s poor solubility are additional factors that contribute to the need for therapeutic adjustments.^8^

Conversely, nanotechnology has provided important advances across various scientific fields, with significant progress reported in pharmacology.^9^ Polymeric nanoparticles represent an alternative for nanomedicine applications. They consist of a continuous polymer matrix capable of entrapping drugs in the core or adsorbing them onto the surface.^10^ These systems stand out as promising carriers for controlled drug release, providing protection against environmental conditions, improving bioavailability, and enhancing therapeutic indices. Furthermore, surface coating allows for higher specificity, making them even more efficient for targeted clinical applications.^10,11^

In this context, nanotechnology-based pharmacological systems have been investigated as a strategy to improve the efficacy of antituberculosis therapy. Different nanocarriers, such as liposomes, polymeric micelles, lipid and polymeric nanoparticles, have been studied for the transport and delivery of antituberculosis drugs, aiming to improve drug bioavailability, stability, and targeted delivery to infection sites. These systems may promote controlled drug release, reduce adverse effects, and increase treatment adherence, thereby contributing to better therapeutic outcomes.^11,43^

In addition, some studies aim to improve the activity, delivery, and cytotoxicity profile of antimicrobial agents recommended for standard treatment, such as rifampicin, considering its antimycobacterial potential. Reports in the literature indicate that nanoformulations containing rifampicin demonstrate greater efficiency in intracellular delivery and contribute to improving activity against *Mycobacterium tuberculosis*.^44^

Among these, poly­(isobutylcyanoacrylate) (PIBCA) has been recognized as a polymer capable of delivering drugs into the cellular cytoplasm, representing a promising candidate for diverse therapies. However, incomplete understanding of the influence of polymer properties and gaps regarding cellular interaction responses have hindered its further development as a drug carrier.^12^

Similarly, fucoidan, a sulfated polysaccharide derived from marine algae, is noteworthy for two major characteristics: its ability not to activate the complement system and its affinity for scavenger receptors (SR), class A types I and II, expressed on the surface of macrophages. These properties make fucoidan effective in extend the half-life of certain drugs and in directing them to specific targets. Therefore, fucoidan holds potential for the development of more efficient and targeted clinical therapies.^12,13,40^

Fucoidan-coated PIBCA nanoparticles have been extensively investigated as drug delivery systems due to their promising properties, including enhanced cellular targeting and broad biomedical applications, such as cancer therapy and antimicrobial strategies.^12,22,41,42^ Despite these advances and their wide range of applications, studies exploring the use of fucoidan-coated PIBCA nanoparticles for the delivery of antituberculosis drugs remain limited. Although previous studies have reported rifampicin-loaded nanosystems^27,47,49^ and fucoidan-functionalized delivery platforms,^12,14,41,42^ studies combining rifampicin-loaded fucoidan-coated PIBCA nanoparticles for antimycobacterial applications against MDR-TB strains are still scarce. Therefore, this nanostructured system may represent a promising strategy to improve rifampicin delivery through fucoidan-mediated macrophage targeting, which may increase intracellular drug accumulation and enhance pharmacological efficacy against both drug-sensitive and multidrug-resistant *Mycobacterium tuberculosis* strains.

Although Mtb is a facultative intracellular microorganism, its replication and infection cycle primarily occur within the host’s pulmonary macrophages.^13^ Previous studies have highlighted the improved performance of PIBCA when coated with fucoidan, enhancing the polymer’s uptake and delivery into macrophages.^12,15^ Accordingly, the present study developed and characterized fucoidan-coated PIBCA nanoparticles loaded with RIF, and evaluated in vitro antimycobacterial activity (MIC), cell viability, and macrophage uptake assays of the formulation against drug-sensitive and multidrug-resistant Mtb strains.

## Materials and Methods

### Fucoidan-Coated PIBCA Nanoparticles

Fucoidan with a molecular weight of 30 kDa was extracted and purified from *Fucus vesiculosus* at the Biochemistry Department of the Keizo-Asami Immunopathology Laboratory, Federal University of Pernambuco (LIKA), by the Nanotechnology, Biotechnology and Cell Culture group (NanoBioCel). Isobutylcyanoacrylate (IBCA) was kindly provided by the same research group. Rifampicin (≥95% purity, HPLC grade; Sigma-Aldrich, St. Louis, MO, USA) was used as the model drug. All other reagents employed in nanoparticle preparation were of analytical grade and used as received.

### Preparation of Nanoparticles

Nanoparticles were prepared by the anionic emulsion polymerization method (AEP) in an acidic aqueous medium, according to previous studies by Lira et al.^12^ A fucoidan solution (1:100 v/v) as stabilizing agents, was prepared by dissolving 0.050 g of fucoidan in 5 mL of water under vigorous stirring at room temperature for 10 min, and the pH was adjusted to 2.5 with 0.1 M HCl. Then, 50 μL of IBCA monomer was added dropwise under constant stirring, initiating the polymerization reaction. The mixture was maintained at room temperature under the same conditions for 3 h; where the acid conditions help the reaction rate and nanoparticle formation. Nanoparticles were transferred into a dialysis bag (Spectro/Por membrane, MWCO 100.000 g/mol, Biovalley, Marne la Vallée, France) and dialyzed against water for 18 h, with four external medium exchanges. RIF-loaded nanoparticles were prepared by adding 0.100 mL of the RIF dissolved in an ethanol:water mixture (2:1 v/v) solution (10 mg/mL) at the beginning of IBCA polymerization. After dialysis, the nanoparticle suspensions were stored at 4 °C until use. Nanoparticles were fluorescently labeled by adding 100 μL of a rhodamine-123 solution in ethanol (5 mg/mL) during preparation.

The stability of each formulation (size and charge variations) was evaluated over 30 days after storage at 4 °C.

### Nanoparticle Characterization

The hydrodynamic diameter (Z-Ave), polydispersity index (PDI), and surface charge (zeta potential) were measured by dynamic light scattering (DLS). Nanoparticle dispersions were diluted in deionized water (1:20 v/v) and analyzed on a Zetasizer SZ90 (Malvern, USA) using a polystyrene cuvette (1 cm^2^) at a fixed angle of 90°. Morphology was examined by scanning electron microscopy (SEM, JSM-5600LV, JEOL) at the Aggeu Magalhães Institute (FIOCRUZ/PE). Samples were placed onto carbon double-sided tape on stubs, dried at room temperature, metalized (LEICA EM ACE 200) after 24 h, and observed under SEM.

To assess morphology after freeze-drying, 1 mL of each nanoparticle suspension was frozen at −80 °C overnight, then lyophilized for 48 h. Dried nanoparticles were mounted on carbon tape and analyzed by SEM as described above.

Chemical interactions among nanoparticle compounds were determined by Fourier-transform infrared spectroscopy (FTIR). Analyses were performed using purified fucoidan, free rifampicin (RIF), lyophilized empty nanoparticles (Nano_Empty), and rifampicin-loaded nanoparticles (Nano_RIF). The spectra were obtained using an FTIR spectrometer (PerkinElmer) within the wavenumber range of 650–4000 cm^–1^. All samples were analyzed in the solid state after lyophilization to evaluate possible interactions between the polymeric matrix, fucoidan coating, and encapsulated rifampicin.

### Determination of Rifampicin Encapsulation Efficiency

RIF quantification was adapted from Swamy et al.^15^ and based on measuring drug absorbance in 0.1 M HCl at 337 nm, using a UV–Vis spectrophotometer (Ultrospec 300 PRO, Amersham Pharmacia Biotech, France). A calibration curve was prepared with RIF standard solutions in different concentrations (1.5–30 μg/mL) diluted in DMSO:0.1 M HCl (5:1 v/v). Encapsulation efficiency (EE%) was determined after drug extraction from nanoparticles. Extraction was performed by disrupting nanoparticles with DMSO (1:100 v/v), vortexed for 5 min, and incubated at 37 °C overnight. The mixture was diluted 1:5 (v/v) in 0.1 M HCl, and absorbance was measured at 337 nm. Drug concentration was calculated from the calibration curve using the formula:

1 $$\mathrm{EE}\%=\frac{{\mathrm{RIF}}_{t}}{{\mathrm{RIF}}_{i}}\times 100$$

where RIF_t_ is the amount of RIF encapsulated in nanoparticles and RIF_i_ is the amount initially added during preparation.

### Microorganisms and Culture

For biological tests, *M. tuberculosis* H37Rv (ATCC 25618) and *M. tuberculosis* H37Ra (ATCC 25177), a reference strain sensitive to all first-line drugs (isoniazid, rifampicin, ethambutol, pyrazinamide), was obtained from the American Type Culture Collection. Additionally, a multidrug-resistant isolate (MDR-TB 551), resistant to isoniazid and rifampicin) was provided by the Central Public Health Laboratory–Dr. Milton Bezerra Sobral (LACEN–PE).

Bacteria were cultured in Middlebrook 7H9 broth (Sigma-Aldrich), supplemented with 10% OADC (oleic acid, albumin, dextrose, catalase), 0.2% glycerol, and 0.05% Tween 80, and incubated for 7–10 days at 37 °C. Colony-forming unit (CFU) counts were performed on Middlebrook 7H10 (Sigma-Aldrich) agar supplemented with 10% OADC and 0.5% glycerol, with incubation for 21–28 days.

### Antimicrobials Used

Rifampicin (≥95% purity, HPLC grade), ethambutol dihydrochloride, levofloxacin, and amikacin were purchased from Sigma-Aldrich (St. Louis, MO, USA) were used as positive controls. Stock solutions (10 mg/mL) were prepared in sterile distilled water or dimethyl sulfoxide (DMSO), depending on solubility, and stored at −20 °C until use.

### Determination of In Vitro Antimycobacterial Activity

Antimycobacterial activity of nanoparticles was determined by the minimum inhibitory concentration (MIC) using the colorimetric microdilution assay in 96-well plates, as described by Palomino et al.^16^ To avoid evaporation during long incubation (up to 15 days), 200 μL of sterile water was added to the outer wells. Serial dilutions (8.25–0.051 μg/mL) of nanoparticles and free RIF were prepared in 7H9 broth supplemented with 10% OADC and 0.2% glycerol, and 100 μL aliquots were distributed into test wells. RIF (8.25 μg/mL) was used as positive control for H37Rv, and amikacin (20 μg/mL) for MDR-TB 551. Control wells included growth and sterility controls.

Bacterial cultures in logarithmic phase were vortexed with glass beads in PBS + 0.05% Tween 80, allowed sediment for 15 min, and the supernatant standardized to McFarland 1.0, followed by a 1:20 dilution (∼1.5 × 10^7^ CFU/mL). 100 μL was added to each well. Plates were sealed with plastic film and incubated at 37 °C for 7 days. After incubation, 30 μL of 0.01% resazurin was added, and plates were incubated overnight at 37 °C. MIC was defined as the lowest concentration preventing the color shift from blue (oxidized) to pink (reduced).

### Cytotoxicity (CC_50_)

Cytotoxicity was evaluated by the thiazolyl blue tetrazolium bromide (MTT; ≥97.5% purity, HPLC grade; Sigma-Aldrich) assay in 96-well plates, following Mosmann et al.^17^ with modifications, using murine macrophage J774A.1 cells (ATCC TIB-67). Cells were cultured in Dulbecco’s Modified Eagle’s Medium (DMEM), high-glucose formulation (4.5 g/L) supplemented with 10% fetal bovine serum (FBS) and 1% antibiotics (streptomycin 100 μg/mL, penicillin 100 U/mL) at 37 °C with 5% CO_2_ until 80% confluence.

Cell viability was determined by Trypan blue exclusion method and adjusted to 1 × 10^5^ cells/well. After 24 h adherence, nanoparticles diluted in medium (8.25–0.051 μg/mL RIF equivalents) were added (100 μL/well) and the vials were incubated for 24 h. Controls included untreated cells, medium with 1% DMSO, and free RIF.

After exposure, 50 μL of MTT solution in PBS (5 mg/mL) was added and incubated for 3 h at 37 °C. Medium was then removed, and 100 μL of DMSO was added to dissolve formazan crystals. Absorbance was measured at 570 nm using a Multiskan FC spectrophotometer (Thermo Scientific, USA).

### In Vitro Drug Interaction Assay

Drug interaction was assessed using the classical checkerboard microdilution method, as described by Lorian et al.^18^ Rifampicin and ethambutol were tested against H37Rv, and amikacin and levofloxacin against MDR-TB 551. Serial dilutions of reference antibiotics were placed along the *x*-axis and nanoparticle dilutions (starting at 8× MIC) along the *y*-axis of 96-well plates. 100 μL of bacterial suspension (1 × 10^7^ CFU/mL) was added to each well. Growth and sterility controls were included. Plates were sealed and incubated at 37 °C for 7 days.

After incubation, 30 μL of 0.02% resazurin solution was added, and plates incubated for an additional 48 h. Results were interpreted using the fractional inhibitory concentration index (FICI), where FIC A = MIC of drug A in combination/MIC of drug A alone. Values of FICI ≤ 0.50 were considered synergistic; >0.50–4.0, indifferent; and >4.0, antagonistic. Synergistic results were represented graphically by isobolograms (Caleffi-Ferracioli et al., 2013).

2 ∑FICI=FICA+FICB

where FIC A = CIM A in the presence of B/CIM A and FIC B = CIM B in the presence of A/CIM B.

### Mycobacterial Killing Kinetics

A standardized inoculum of approximately 5 × 10^7^ CFU/mL of *M. tuberculosis* H37Rv in logarithmic growth phase was resuspended in Middlebrook 7H9 broth supplemented with 10% OADC and 0.2% glycerol. Aliquots of this suspension were distributed into 50 mL conical tubes containing 7H9 broth under the following conditions: control (no inhibitor); 1× MIC of RIF; and 1× MIC of nanoparticles. All tubes were incubated at 37 °C with agitation at 120 rpm, by Lorian et al.^18^ To quantify the number of viable bacteria over time, 100 μL samples were collected from each tube starting at time 0 h, followed by serial 10-fold dilutions in PBS containing 0.05% Tween 80. Aliquots were collected at 5, and 10 days. Dilutions were plated on Middlebrook 7H10 agar supplemented with 10% OADC and 0.5% glycerol and incubated at 37 °C with 5% CO_2_ for 21–28 days (Garima et al., 2015). After this period, CFUs were counted and results plotted as log_10_ CFU versus time. Experiments were performed in triplicate.

### In Vitro Infection Model

Infection of J774A.1 macrophages with *M. tuberculosis* was performed according to Santos et al.,^19^ with modifications. Prior to infection, bacilli were cultured for 2 days at 37 °C in 7H9 broth. On the day of the experiment, the inoculum was standardized to McFarland 1.0. The suspension was passed ten times through a 27G insulin needle to obtain a predominantly unicellular bacterial suspension, then diluted in DMEM to yield an inoculum of approximately 5 × 10^6^ CFU/mL.

J774A.1 Cells were seeded into 96-well plates at 1 × 10^5^ cells/well and incubated for 24 h at 37 °C with 5% CO_2_. After this period, the supernatant containing nonadherent cells was removed, and wells were washed twice with PBS. Cells were then infected with the bacterial suspension in supplemented DMEM at a multiplicity of infection (MOI) of 10:1. After 4 h of infection, the supernatant was removed, and wells were washed three times with PBS. Nanoparticles were diluted in DMEM at concentrations corresponding to 2× MIC, 1× MIC, and 0.5× MIC, and added to the wells.

At time zero postinfection, triplicate wells were lysed with 0.025% SDS to recover intracellular bacilli. Control wells received supplemented DMEM with 0.2% DMSO (vehicle). Plates were incubated at 37 °C with 5% CO_2_. In addition to time zero, wells were analyzed at 1, 3, and 5-days postinfection. Aliquots (100 μL) of each lysate were diluted in PBS and plated on 7H10 agar supplemented with 10% OADC. Plates were incubated at 37 °C for 21–28 days, followed by CFU counting.

### Cellular Uptake Assay

Murine macrophage J774A.1 cells (ATCC TIB-67) were cultured in Dulbecco’s Modified Eagle’s Medium (DMEM), high-glucose formulation (4.5 g/L) supplemented with 10% fetal bovine serum (FBS) and 1% antibiotics (streptomycin 100 μg/mL, penicillin 100 U/mL) at 37 °C with 5% CO_2_ until 80–90% confluence. Cells were counted using Trypan blue exclusion and seeded at 1.5 × 10^6^ cells/well in 6-well plates, followed by incubation for 24 h under the same conditions for macrophage adhesion.

Inocula of *M. tuberculosis* H37Rv and MDR-TB (551) adjusted to 1 × 10^6^ CFU/mL (McFarland 1.0) were passed through a 27G needle and resuspended in supplemented DMEM. After washing adhered macrophages twice with PBS, inocula were added, and plates incubated for 4 h at 37 °C with 5% CO_2_ to allow phagocytosis.

Cells were then treated with Nano_RIF and Rhodamine 123 (R8004, fluorescent dye; Sigma-Aldrich, St. Louis, MO, USA) stock solution was prepared in ethanol according to manufacturer’s recommendations and diluted to the working concentration immediately before use. Nano_RIF and Rhodamine 123 in supplemented DMEM at a RIF-equivalent concentration of 0.206 μg/mL (2× MIC for the inoculum used). At 1, 2, and 3 h, wells were washed twice with PBS, and cells detached with a cell scraper, transferred to microtubes, and centrifuged at 2000 rpm for 5 min. Supernatants were discarded, pellets resuspended in 500 μL of paraformaldehyde, and analyzed by flow cytometry (FACS Calibur, Becton Dickinson). At least 30.000 events per tube were acquired. Analysis was performed by gating cells according to size (FSC) and granularity (SSC), and mean fluorescence intensity was measured in channel FL1 (BP 530/30).

### Statistical Analysis

All experiments were performed in triplicate and repeated in three independent experiments. Data are presented as mean ± standard deviation (SD). Colony-forming unit (CFU) values were log_10_-transformed prior to analysis. Data normality and homogeneity of variances were assessed using appropriate tests. Statistical analyses were performed using GraphPad Prism software (version 8.0.2; GraphPad Software, San Diego, CA, USA). Depending on the experimental design, comparisons between two groups were performed using Student’s *t* test, while multiple-group comparisons were analyzed using one-way or two-way ANOVA followed by Tukey’s, Dunnett’s, or Šídák’s multiple comparisons tests, as indicated in each figure legend. Exact p-values and significance levels are presented in the corresponding figures and legends. Differences were considered statistically significant at *p* < 0.05.

## Results and Discussion

### Preparation and Characterization of PIBCA Nanoparticles

The average particle size was below 400 nm (Figure 1A), with a narrow size distribution, presenting a polydispersity index (PDI) of 0.2. Zeta potential analysis confirmed the presence of fucoidan on the surface of the polymeric nanoparticles produced (Figure 1B). According to studies by Zandanel et al.^20^ and Mogoşanu et al.,^21^ these properties are associated with a formulation displaying colloidal stability and enhanced interaction with scavenger-type macrophage receptors, which can recognize highly negatively charged components.

**Figure 1:**
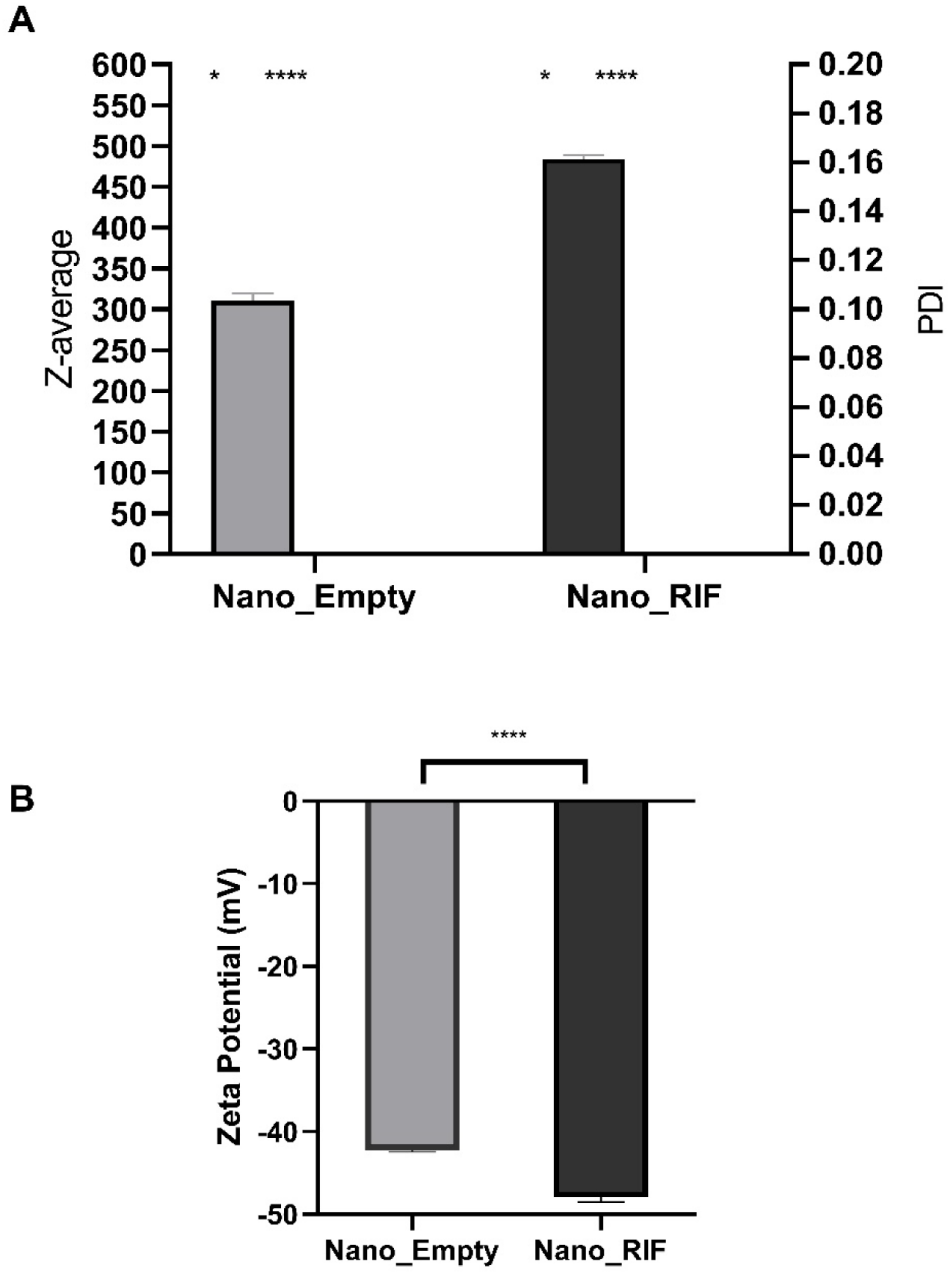
Comparison of (A) Z-average (nm) and polydispersity index (PDI) and (B) zeta potential (mV) of fucoidan-coated PIBCA nanoparticles without drug (Nano_Empty) and loaded with rifampicin (Nano_RIF). Samples were diluted (1:20) and analyzed in triplicate using a Zetasizer SZ90 (Malvern, USA). Data are expressed as mean ± standard deviation (SD) from three independent experiments (*n* = 3). Statistical analysis was performed using two-way ANOVA. Significant differences were considered at **** *p* < 0.0001.

Furthermore, the mean diameter of approximately 400–480 nm is compatible with macrophage internalization mechanisms. Studies such as those by Rudolph et al.^45^ report that macrophages are capable of internalizing particles ranging from the submicrometric scale to several micrometers through phagocytosis, where demonstrated efficient uptake of particles up to 6 μm by macrophage cell lines such as J774A.1.

Additionally, studies conducted by Feito et al.^46^ emphasize that biomaterial particles and nanospheres can efficiently interact with macrophages without compromising cellular function. These observations are consistent with our experimental findings, in which efficient intracellular activity was observed in infected macrophages, indicating that particle sizes larger than those observed in our study do not prevent cellular internalization. Considering that *Mycobacterium tuberculosis* primarily resides within macrophages, particle sizes within this range may favor phagocytic uptake and intracellular drug delivery, which are important features for tuberculosis-targeted therapies

The encapsulation of rifampicin remains a significant challenge in the development of nanostructured delivery systems, mainly due to the physicochemical characteristics of the drug, including its instability under certain conditions, tendency to degradation, and limitations related to its interaction with different polymeric matrices. In polysaccharide-based polymeric systems, such as dextran, chitosan, and fucoidan, the literature frequently describes moderate encapsulation rates, generally ranging from 40 to 70%,^22^ or reports only physicochemical properties and colloidal stability without thoroughly addressing encapsulation efficiency.^20^ In addition, higher encapsulation efficiencies previously reported for rifampicin-loaded systems are often associated with conventional polymeric formulations that do not include surface functionalization or polysaccharide coatings, which may increase formulation complexity and directly affect drug entrapment efficiency.^47^

The encapsulation efficiency of rifampicin, calculated using the calibration curve equation obtained from RIF standards, showed moderate values, with an average of approximately 50% ± 10%, remaining within the range commonly reported for polysaccharide-based nanosystems. Although some studies involving rifampicin drug delivery systems have reported encapsulation efficiencies close to 70%, these results vary considerably depending on the polymer-to-drug ratio, the preparation method employed, and the chemical interactions established between the formulation components.^47^

Therefore, the results obtained in the present study demonstrate a performance consistent with that observed for nanoparticulate systems designed for rifampicin delivery, reinforcing both the feasibility and originality of the developed formulation, especially considering the expected behavior of polysaccharide-coated poly­(alkylcyanoacrylate) nanoparticles.

After 30 days of storage at 4 °C, size variations were observed for Nano_Empty (Figure 2A) and Nano_RIF (Figure 2C). In contrast, RIF-loaded nanoparticles maintained a consistent size range throughout the entire analysis period. Minimal variations were detected in zeta potential values (Figure 2B and 2D). These results are consistent with the review by Hamouda et al.,^22^ which reported stable formulations of systems employing fucoidan and other sulfated polysaccharides as stabilizers in nanostructures, since polysaccharides confer increased electrostatic repulsion and surface protection.

**Figure 2:**
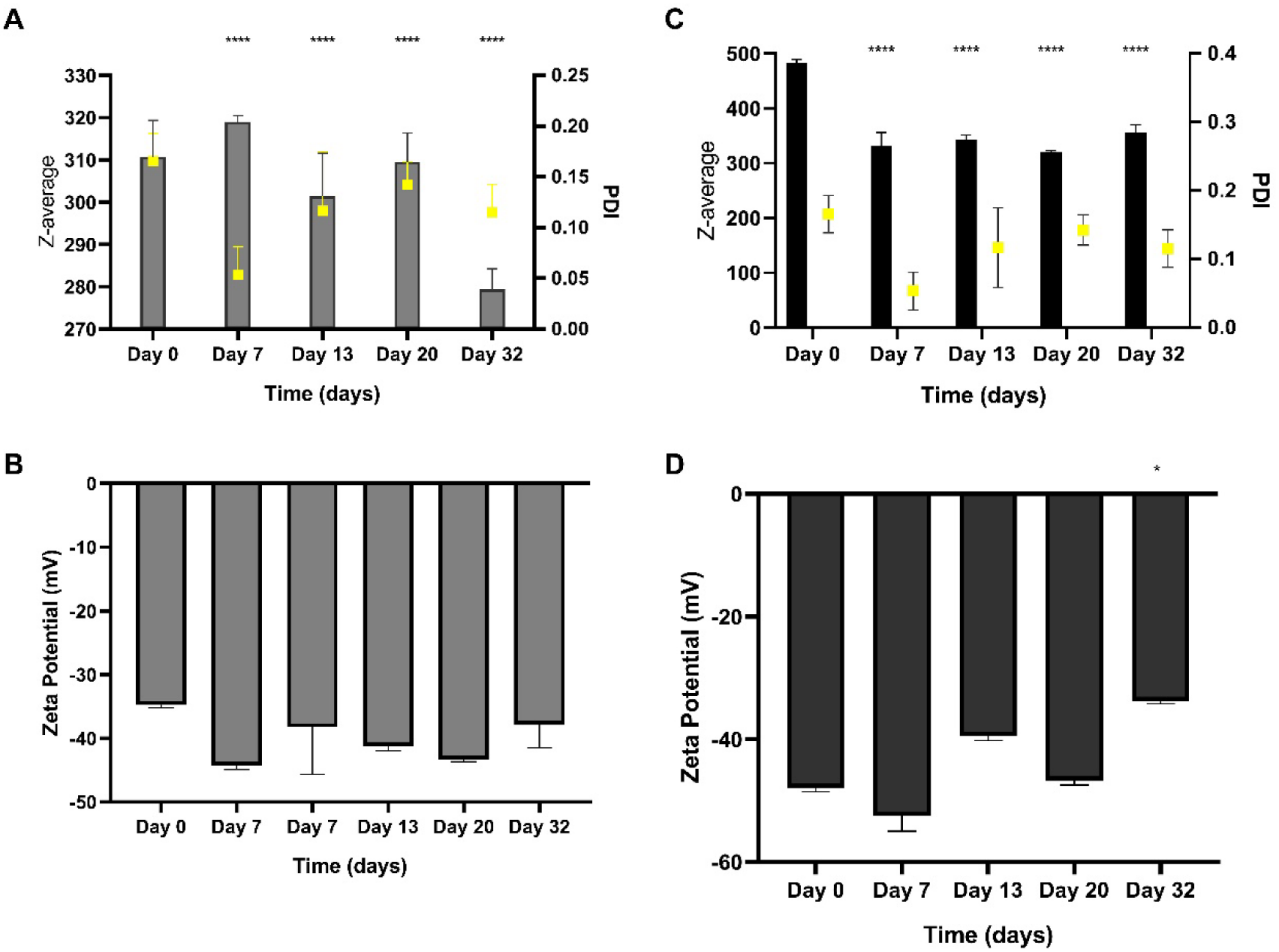
Stability evaluation of fucoidan-coated PIBCA nanoparticles stored at 4 °C. (A) Z-average (hydrodynamic diameter) and polydispersity index (PDI) of empty nanoparticles (Nano_Empty). (B) Z-average (hydrodynamic diameter) and PDI of rifampicin-loaded nanoparticles (Nano_RIF). (C) Zeta potential of Nano_Empty. (D) Zeta potential of Nano_RIF. Samples were diluted 1:100 and analyzed in triplicate using a Zetasizer SZ90 (Malvern, USA). Data are expressed as mean ± standard deviation (SD) from three independent experiments (*n* = 3). Statistical analysis for hydrodynamic diameter and PDI was performed using two-way ANOVA, while zeta potential data were analyzed using multiple *t* tests. Significant differences were considered at * *p* < 0.05 and **** *p* < 0.0001.

Dispersion of nanoparticles in water and subsequent lyophilization did not cause alterations in particle size or morphology, as observed in Figure 3. Although Zandanel et al.^20^ and Cavalcanti et al.^42^ did not perform lyophilization assays, they emphasized that PIBCA nanoparticles coated with polysaccharides exhibit colloidal stability, which may be related to the results observed in this assay.

**Figure 3:**
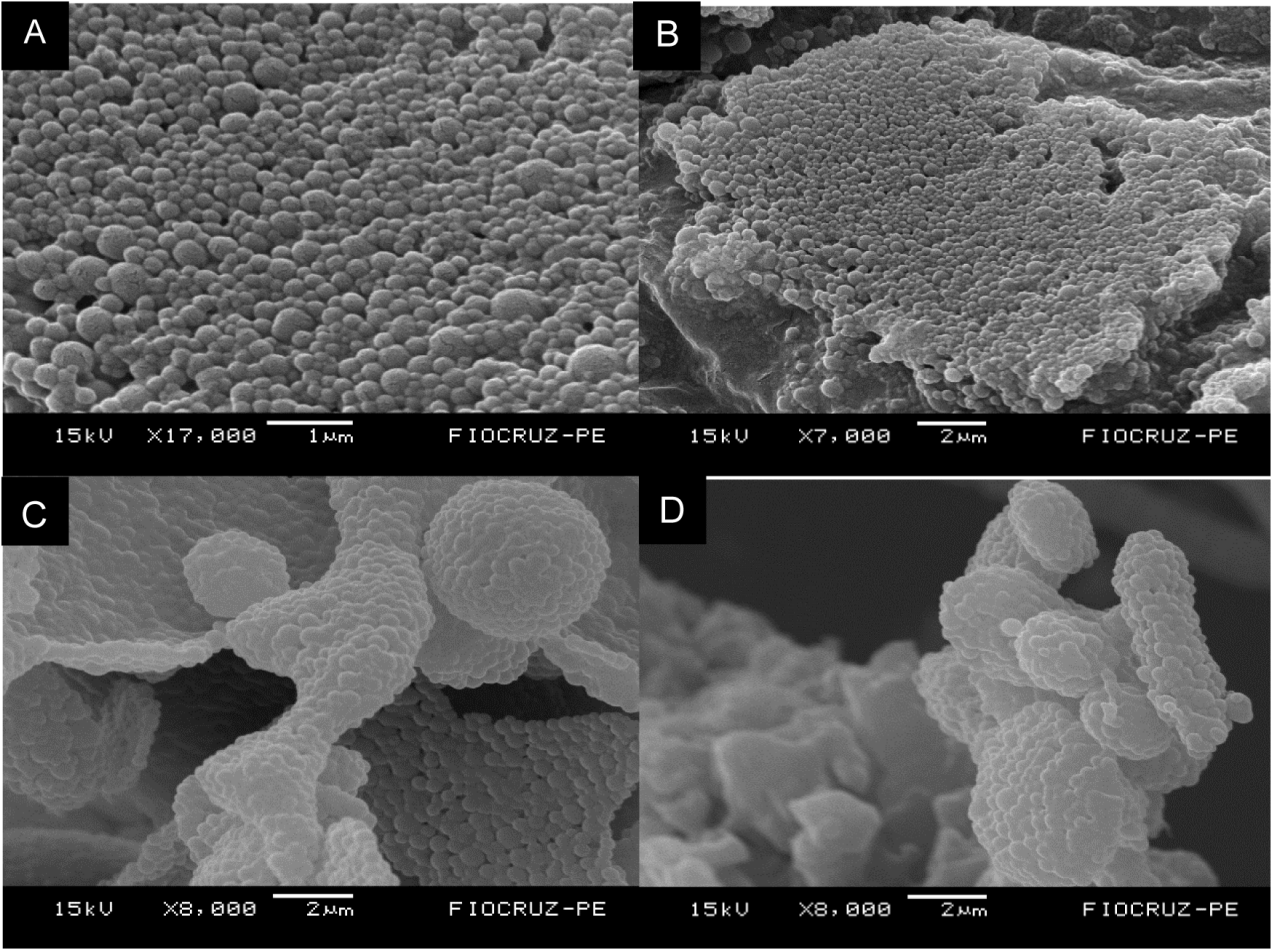
Morphology and distribution of fucoidan-coated PIBCA nanoparticles analyzed by scanning electron microscopy. Liquid formulations: (A) Nano_Empty and (B) Nano_RIF. After lyophilization: (C) dried Nano_Empty and (D) dried Nano_RIF.

The FTIR (Figure 4) spectrum of pure rifampicin exhibited characteristic bands associated with hydroxyl groups (O–H) in the region of 3200–3500 cm^–1^, as well as bands corresponding to C=O bonds near 1700 cm^–1^ and fingerprint region signals between 1000 and 1600 cm^–1^, consistent with previous reports in the literature.^50,51^ The fucoidan spectrum presented characteristic sulfate group (S=O) bands in the region of 1200–1260 cm^–1^, which are commonly associated with sulfated polysaccharides.^52^

**Figure 4:**
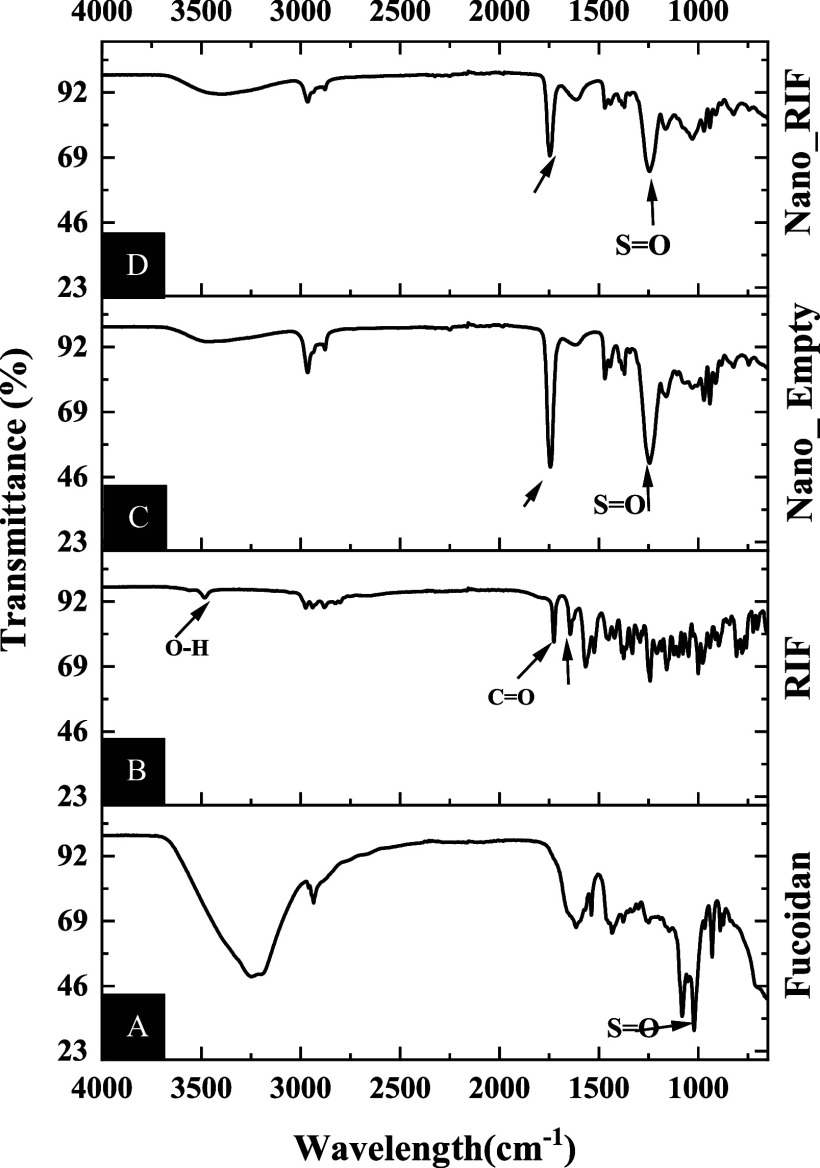
FTIR spectra of fucoidan (a), rifampicin (RIF) (b), empty nanoparticles (Nano_Empty) (c), and rifampicin-loaded nanoparticles (Nano_RIF) (d) in the range of 650–4000 cm^–1^.

In the Nano_RIF formulation, it was possible to observe displacement and/or reduction in the intensity of characteristic fucoidan bands (S=O) in the 1200–1260 cm^–1^ region, suggesting interactions between rifampicin and the produced nanoparticle system. These spectral modifications may indicate intermolecular interactions between rifampicin, fucoidan, and the PIBCA matrix, reinforcing the successful incorporation of the drug into the nanosystem. Similar spectral profile alterations after drug incorporation into polymeric nanostructures have been previously described in the literature.^51^

### Antimycobacterial Activity (MIC) and Cytotoxicity (CC_50_)

Nanoparticles were evaluated against drug-sensitive *M. tuberculosis* strains (H37Ra; H37Rv and MDR-TB (551)). The aim was to assess the efficacy of the nanoparticle formulation compared with the encapsulated pharmaceutical agent (RIF), particularly against the MDR-TB strains.

The formulations exhibited antimycobacterial activity against both the drug-sensitive *M. tuberculosis* strains and the multidrug-resistant clinical isolate (Table 1). Inhibitory concentrations ranged from 0.103 μg/mL to 0.412 μg/mL for the sensitive strains and 1.238 μg/mL for MDR-TB strain. Although the nonencapsulated drug showed lower MIC values than the nanoparticle formulation against the sensitive strain, the efficacy demonstrated by Nano_RIF against the multidrug-resistant isolate suggests that RIF encapsulation may improve antimicrobial activity against resistant strains associated with rifampicin. Previous studies, such as those by Yessentayeva et al.,^48^ have demonstrated that rifampicin-loaded nanostructured systems can enhance antimycobacterial activity, particularly in intracellular models.

**Table 1:** Antimycobacterial Activity of Nano_RIF and Free RIF against Drug-Sensitive and Multidrug-Resistant *M. tuberculosis* Strains, Expressed as Minimum Inhibitory Concentration (MIC)^a^

	MIC (μg/mL)		
sample	H37Ra	H37Rv	MDR-TB (551)
Nano_RIF	0.103	0.412	1.238
RIF	<0.051	0.103	>8.25
Nano_Empty	>8.25	>8.25	>8.25

a Abbreviations: MIC, minimum inhibitory concentration; Nano_Empty, fucoidan-coated PIBCA nanoparticle without drug; RIF, rifampicin; Nano_RIF, fucoidan-coated PIBCA nanoparticle loaded with RIF.

H37Ra, H37Rv and drug-resistant clinical isolates such as MDR-TB strains exhibit phenotypic and genotypic differences that directly affect their virulence and growth profiles. Stanley et al.^23^ and Mashabela et al.^24^ reported that H37Ra is an attenuated variant of H37Rv, characterized by reduced intracellular replication capacity and decreased expression of essential virulence factors, such as inactivation of ESAT-6 (Early Secreted Antigenic Target 6) secretion, which is associated with metabolically active mycobacteria. In contrast, Stanley et al.,^23^ Mashabela et al.,^24^ and Drysdale et al.^25^ indicated that H37Rv retains its infection and immune evasion capacity, which directly translates into higher bacterial loads and accelerated in vitro and in vivo growth.

Goossens et al.,^5^ Drysdale et al.,^25^ and Xu et al.^26^ reported that drug-resistant clinical isolates maintain, and in some cases even enhance, their virulence capacity through compensatory mutations and metabolic reprogramming, in addition to drug-associated genetic mutations. Rifampicin resistance remains incompletely understood; however, according to Goossens et al.^5^ and Xu et al.,^26^ it predominantly arises from genetic mutations in the *rpoB* gene, which alter the binding site on the β-subunit of RNA polymerase (the site of RIF action), reducing the drug’s affinity.

These characteristics contribute to the requirement for higher drug concentrations for inhibition, as observed in the higher MIC values for the MDR-TB strain in this study. However, while free RIF outperformed Nano_RIF in less virulent strains, the nanoparticle system demonstrated an advantage against the MDR-TB strain. The lack of activity of free RIF (MIC > 8.25 μg/mL) compared to the maintained efficacy of Nano_RIF (MIC = 1.238 μg/mL) suggests that nanoparticle encapsulation may improve rifampicin activity against resistant strains, possibly by enhancing intracellular delivery, protecting the drug from degradation, and increasing local drug concentration within macrophages. This advantage, as highlighted by Drysdale et al.^25^ and Ali et al.,^27^ may be associated with optimized intracellular drug delivery by the nanoparticle system, which can maintain inhibitory levels even under conditions of high virulence and resistance.

The use of fucoidan-coated PIBCA nanoparticles offers strategic advantages in this context. The biodegradable polymer allowed efficient RIF encapsulation, while the polysaccharide coating favored recognition by receptors present on macrophages. According to Ali et al.,^27^ these factors may enhance internalization and drug delivery at the infection site.

Cytotoxicity was assessed using the MTT reduction assay (3-(4,5-dimethylthiazol-2-yl)-2,5-diphenyltetrazolium bromide) in J774A.1 macrophages. The MTT assay is widely used to quantify cell proliferative activity by measuring the conversion of MTT by mitochondrial dehydrogenases in metabolically active cells.^28^ Cell viability was evaluated for both nanoparticles (Nano_Empty and Nano_RIF) and free RIF.

The J774A.1 cell line, a murine macrophage cell line of mammalian origin, is widely used as a model to assess immunotoxicity, phagocytosis, and inflammatory response, as described according to Khatua et al.^28^ Moreover, Gudgeon et al.^29^ reported that J774A.1 cells exhibit a proteomic profile like primary bone marrow-derived macrophages, particularly in the expression of proteins involved in phagocytosis and immune response. Additionally, they display a higher bacterial internalization capacity than other cell lines, such as RAW264.7 and BMA3.1A7, making them a functional model for infection and uptake studies.

As shown in Figure 5, the Nano_RIF formulation-maintained cell viability above 50% at all tested concentrations. Only the highest concentration tested (3 μg/mL) reduced cell viability by approximately 40%. According to Singh et al.,^31^ compounds or molecules that maintain ≥50% cell viability are considered nontoxic. This low-toxicity profile was also reported by Lemos et al.^30^ when testing other bioactive formulations in murine macrophages. These findings indicate that Nano_RIF exhibits low cytotoxicity and is biocompatible with mammalian cells, particularly macrophages. Considering that macrophages are the primary host cells of *Mycobacterium tuberculosis*, maintaining their viability is essential to ensure the safety and applicability of the proposed drug delivery system.

**Figure 5:**
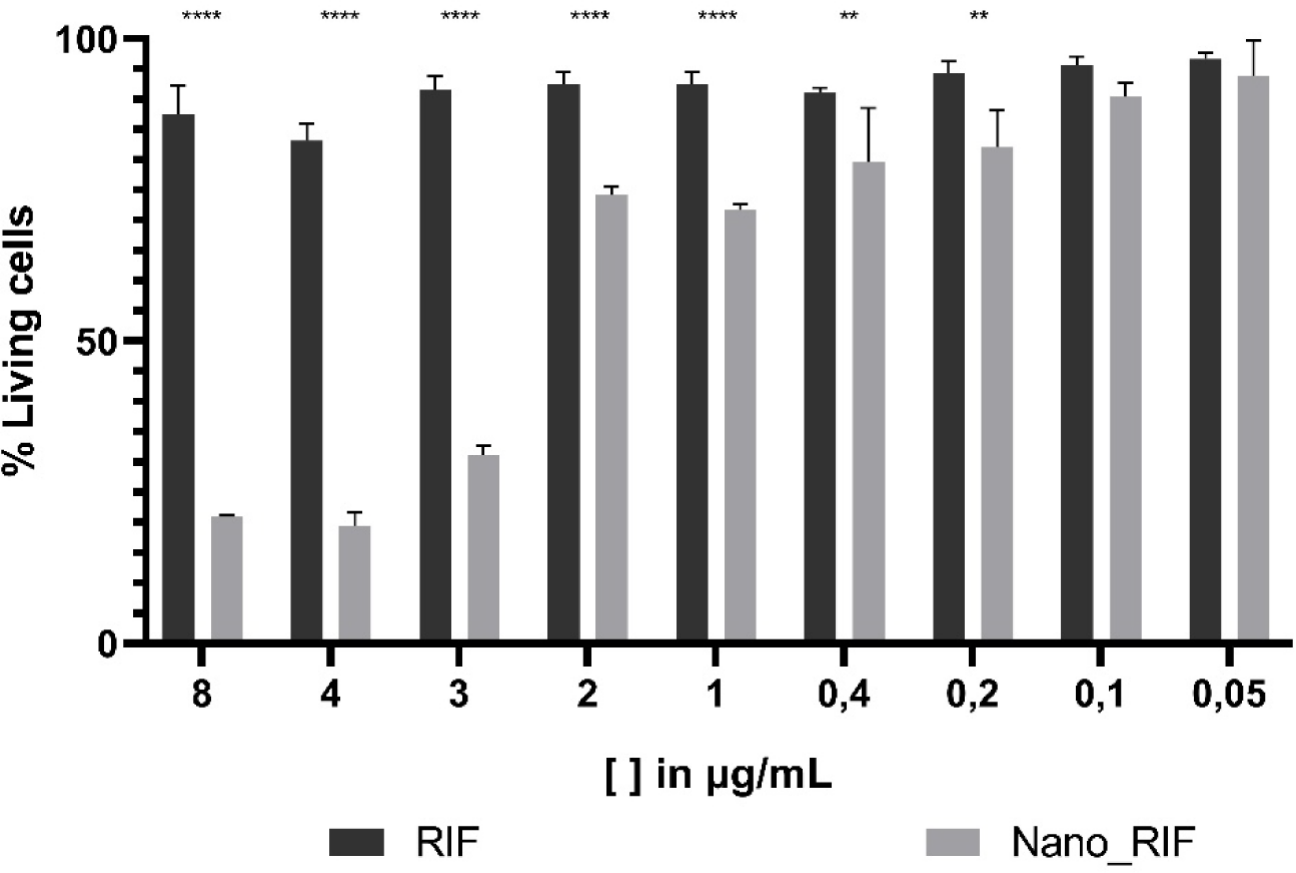
Cytotoxicity assessment of nanoformulated rifampicin (Nano_RIF) and free rifampicin (RIF) in J774A.1 cells by MTT assay after 24 h of exposure. Cell viability (%) is expressed as mean ± standard deviation (SD) from three independent experiments (*n* = 3). Statistical analysis was performed using two-way ANOVA followed by Šídák’s multiple comparisons test. Significant differences between treatments at the same concentration were considered at **p < 0.01 and ****p < 0.0001.

When analyzing both antimycobacterial inhibition values and cell viability (Table 2), it becomes evident that the concentrations tolerated the cells were higher than those required to inhibit the growth of both drug-sensitive and drug-resistant Mtb. Therefore, the developed Nano_RIF formulation did not exhibit cytotoxic effects on cells, particularly at concentrations necessary to inhibit the bacterial agent. This finding is consistent with Gudgeon et al.^29^ and Singh et al.,^31^ who described safe murine cell assays in which maintaining viability ≥50% at concentrations near or above the MIC is considered indicative of low cellular toxicity. This finding reinforces the safety of the formulation for biological applications.

**Table 2:** Minimum Inhibitory Concentration (MIC) and Percentage of Viable Cells (CC_50_) against Drug-Sensitive (*M. tuberculosis* H37Ra and H37Rv) and Multidrug-Resistant (MDR-TB) Strains^a^

strain	MIC (μg/mL)	CC_50_ (%)
H37Ra	0.103	90
H37Rv	0.412	80–90
MDR-TB	1.238	70–88

a Abbreviations: MIC, minimum inhibitory concentration; CC_50_, cytotoxic concentration 50; Nano_RIF, fucoidan-coated PIBCA nanoparticle loaded with RIF.

### In Vitro Drug Interaction Determination

To determine the type of pharmacological interaction between Nano_RIF and first-line antimicrobials (rifampicin and ethambutol) as well as second-line drugs (amikacin and levofloxacin), the two-dimensional checkerboard method in 96-well plates was employed. Using the fractional inhibitory concentration index (FICI), it was possible to classify the interaction between molecules as synergistic, additive, or antagonistic.

Among the interactions between the nanoparticle and the antimicrobials, synergistic effects were observed for all first- and second-line drugs tested. The FICI for Nano_RIF ranged from 0.30 to 1.21 for all drugs in the susceptible strain (H37Ra). In MDR-TB, values ranged from 0.48 to 0.40 (Table 3), indicating that Nano_RIF, like the free drug, does not interfere with the action of the main antibiotics used in tuberculosis treatment.

**Table 3:** In Vitro Fractional Inhibitory Concentration of Nano_RIF in Combination with First- and Second-Line Antimicrobials against *M. tuberculosis* H37Rv and MDR-TB Strains^a^

		H37Rv		MDR-TB	
nanoparticle	antimicrobial	combination (FIC A/FIC N)	FIC index	combination (FIC A/FIC N)	FIC index
Nano_RIF	RIF	0.125/0.412	0.30	*/1238	*
	EMB	0.500/0.412	1.21	1.00/2062	0.48
	AMK	0.125/0.412	0.30	0.500/1238	0.40
	LFX	0.500/1.238	0.40	0.500/1238	0.40

a Abbreviations: RIF, rifampicin; EMB, ethambutol; AMK, amikacin; LFX, levofloxacin; FIC, fractional inhibitory concentration; * indicates that RIF showed no activity against the drug-resistant strain.

Tuberculosis treatment relies on the administration of multiple drugs acting together to enhance therapeutic efficacy against Mtb.^32^ The findings of the present study, as highlighted by Khoshnood et al.^33^ and Jubilee et al.,^34^ suggest that this approach represents a promising strategy to potentiate tuberculosis therapy, particularly in cases of drug resistance where effective therapeutic options are more limited.

### Killing Kinetics

The activity of Nano_RIF against the susceptible H37Rv strain, compared with nonencapsulated RIF, was evaluated over a 10-day period, showing a time-dependent reduction in colony-forming units (CFUs) (Figure 6), with 43% inhibition on day 5 and 99.9% elimination of CFUs after the exposure period. These results were similar to those obtained with free RIF.

**Figure 6:**
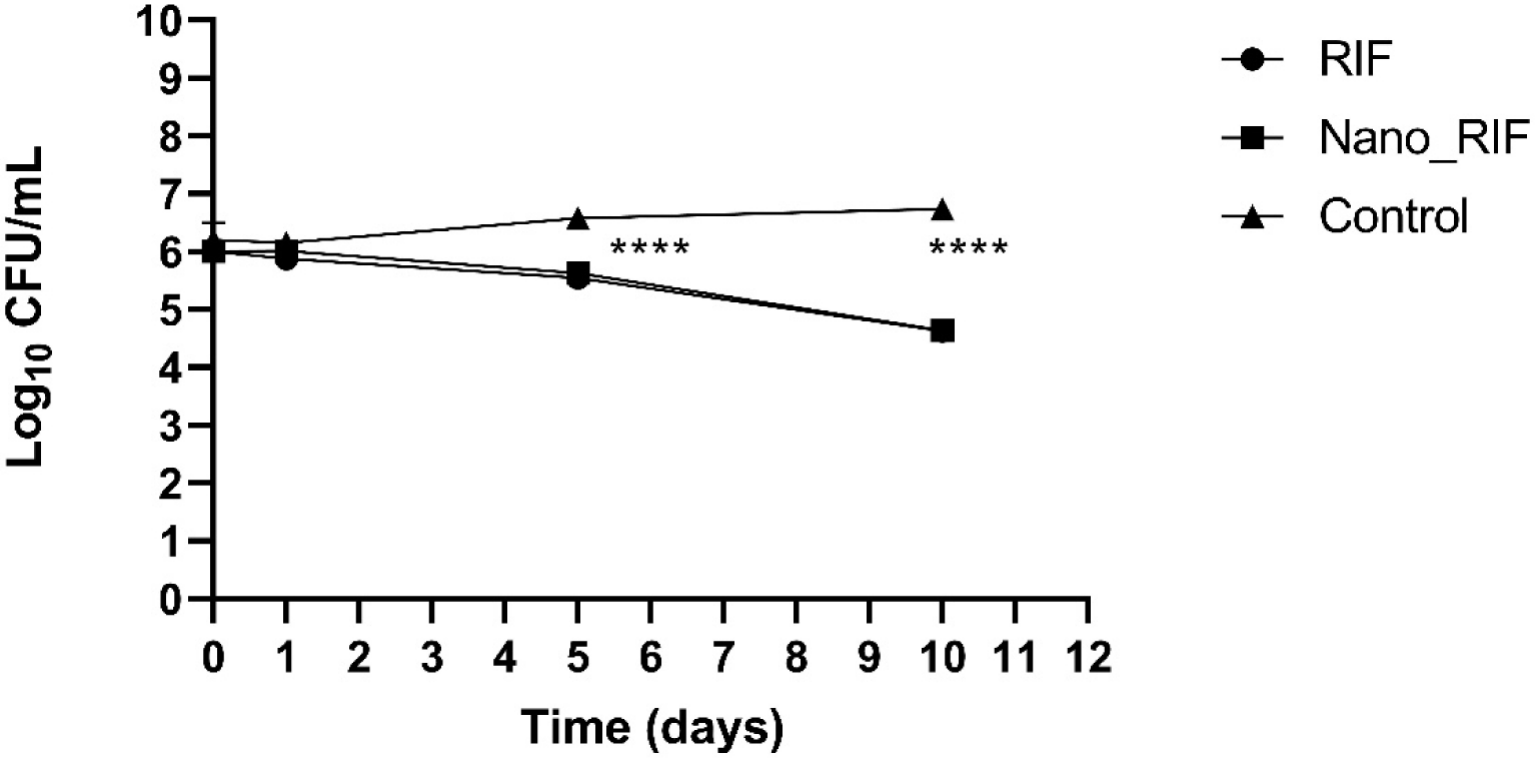
Killing kinetics of Nano_RIF and free rifampicin against drug-sensitive *Mycobacterium tuberculosis* H37Rv. Bacterial viability was expressed as log_10_ CFU/mL over time (0, 1, 5, and 10 days). Treatments were evaluated at a concentration of 0.412 μg/mL. Data are presented as mean ± standard deviation (SD) from three independent experiments (*n* = 3). Statistical analysis was performed using two-way ANOVA followed by Tukey’s multiple comparisons test. Significant differences were considered at **** *p* < 0.0001 compared with the control group.

According to Ishak et al.,^35^ RIF is recognized for its bactericidal profile, a characteristic also demonstrated by Nano_RIF in the present results, indicating that the drug’s bactericidal potential was maintained after the encapsulation process, and confirming the capacity of the produced nanoparticle as an antimicrobial agent. Furthermore, as emphasized by Ishak et al.,^35^ these findings are consistent with the criteria for minimum bactericidal concentration (MBC) in killing kinetics assays, which evaluate the progressive reduction of CFUs over time, in contrast to bacteriostatic antibiotics that merely delay or suppress bacterial replication.

The study by Kim et al.^36^ further highlights the importance of killing kinetics curves for understanding the antimicrobial behavior of antibiotics when in contact with mycobacteria. For example, in the assays of Linhares et al.,^37^ candidate compounds demonstrated gradual, time-dependent reductions comparable to reference drugs against mycobacteria. Such findings consolidate the use of this type of assay in the evaluation of novel pharmacological systems against mycobacteria.

### Intracellular Antimycobacterial Activity

Based on the MIC, cytotoxicity, and killing kinetics results, Nano_RIF was further evaluated for its intracellular activity against *M. tuberculosis*. For this analysis, the J774A.1 cell line was used infected separately with susceptible MDR-TB strains of the bacillus, and treated with the nanoparticle at concentrations of 2-fold below, 1-fold, and 2-fold above the MIC.

In Figure 7A, Nano_RIF applied to susceptible strain showed a similar CFU reduction profile when compared with free drug treatment (Figure 7B). As expected, the lowest concentrations tested (below the MIC) did not promote CFU reduction over time. Conversely, at concentrations of 0.824 μg/mL (Nano_RIF) and 0.412 μg/mL (free RIF), a 99.99% reduction in CFUs was observed over the five-day evaluation period.

**Figure 7:**
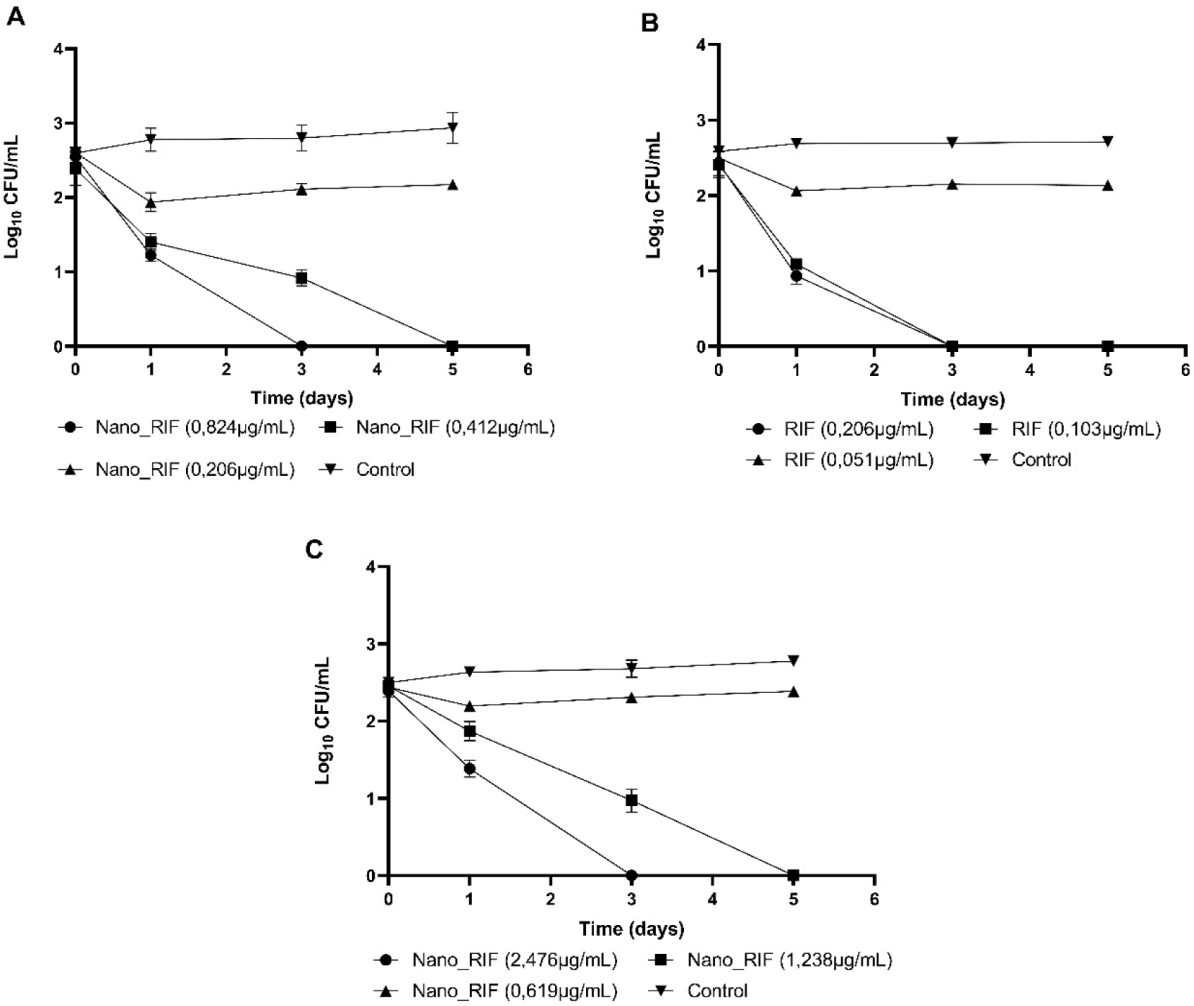
Intracellular antimycobacterial activity in J774A.1 macrophages infected with *Mycobacterium tuberculosis*. Bacterial burden was quantified as log_10_ CFU/mL over time (0, 1, 3, and 5 days). (A) Activity of Nano_RIF against the drug-sensitive H37Rv strain. (B) Activity of free rifampicin (RIF) against the drug-sensitive H37Rv strain. (C) Activity of Nano_RIF against a multidrug-resistant clinical isolate of *M. tuberculosis* (MDR-TB). Data are presented as mean ± standard deviation (SD) from three independent experiments (*n* = 3). Statistical analysis was performed using two-way ANOVA. Significant differences were considered at *p* < 0.05.

It is important to note that, although free RIF also achieved 99.99% inhibition at concentrations of 2-fold above and 1-fold the MIC (0.206 μg/mL and 0.103 μg/mL) starting from day three, one must consider that Nano_RIF requires additional time for complete drug release into the medium before exerting its action on the microorganism.

In addition, Birk et al.^38^ and Guo et al.^39^ reported that polymeric nanoparticles can protect drugs from degradation, promote controlled release, and favor accumulation of the active substance inside macrophages, thereby enhancing antimicrobial activity against mycobacteria. Furthermore, according to Goossens et al.,^5^ the maintenance of Nano_RIF’s bactericidal profile against H37Rv in the intracellular environment reflects the ability of the formulation to adapt and act in the phagocytic milieu.

For the MDR-TB strain, Nano_RIF at a concentration 2-fold below the MIC (0.619 μg/mL) did not show statistically significant CFU reduction (Figure 7C). However, at 2-fold above the MIC (2.476 μg/mL), a 99.99% reduction in colonies was already observed by day three. At 1.238 μg/mL (equivalent to 1-fold the MIC), inhibition ranged from 32.69% on day one to 60.22% on day three, reaching 99.99% CFU reduction by the end of the five-day observation period.

Xu et al.^26^ emphasized that drug-resistant strains such as MDR-TB often harbor resistance mutations accompanied by compensatory mechanisms that preserve—and in some cases even intensify—their virulence, making their eradication inside macrophages even more challenging.

Consequently, we can infer that even under macrophage conditions, the nanoparticle was able to efficiently deliver the drug, maintaining bactericidal inhibition against both the susceptible and resistant strains. Moreover, it is important to highlight that MDR-TB is a clinical isolate resistant to RIF, the drug encapsulated in the formulation. Thus, we suggest that the encapsulation process may contribute to improved activity against resistant strains, while preserving the biological efficacy and activity of the antibiotic.

### Uptake Kinetics

The affinity of fucoidan-coated PIBCA nanoparticles for scavenger receptors (SRA) on macrophages has been well described in the literature.^12,15^ Therefore, to confirm the entry of Nano_RIF into macrophages, a cellular uptake assay was performed and analyzed by flow cytometry. For this purpose, Nano_RIF labeled with rhodamine-123 was incubated with J774A.1 macrophages infected with the drug-resistant Mtb strain (MDR 551).

The assay was performed at 1, 2, and 3 h time points to evaluate phagocytosis of Nano_RIF by infected J774A.1 cells. As shown in Figure 8, the nanoparticles were internalized or fully phagocytosed after 3 h of exposure.

**Figure 8:**
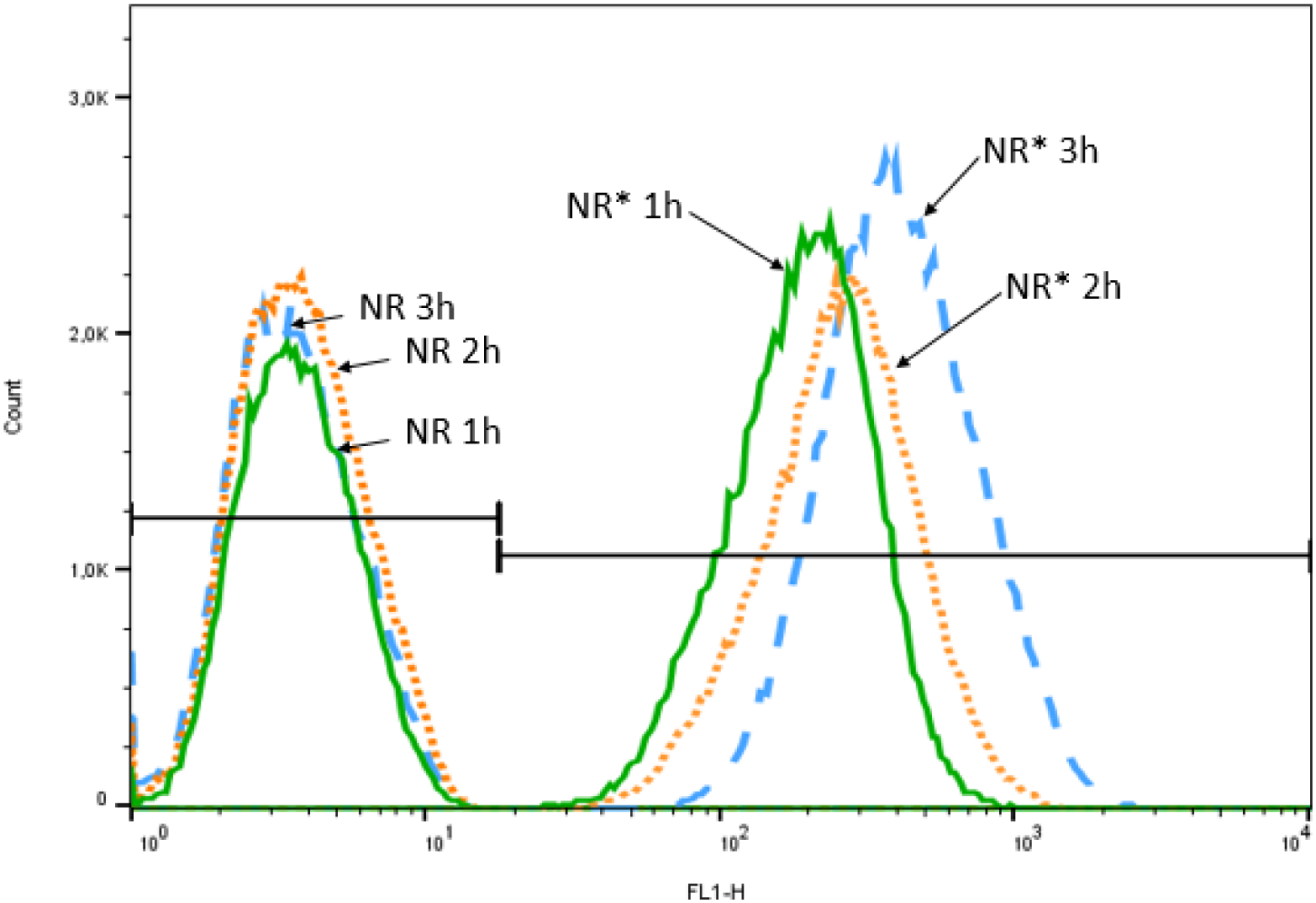
Flow cytometry analysis of the uptake of rhodamine-123-labeled Nano_RIF by J774 macrophages. NR: Nano_RIF; NR*: Nano_RIF labeled with rhodamine-123. Data are presented as mean ± standard deviation (*n* = 3).

Efficient internalization of nanostructured systems by macrophages is frequently reported in the literature. Reviews by Zhang et al.^41^ and Goossens et al.^5^ emphasize that biodegradable polymers and polysaccharides exhibit properties that facilitate adhesion and cellular uptake, promoting higher drug concentrations within the phagolysosomal compartment. Furthermore, the study by Guo et al.^39^ demonstrated that polymeric systems can be engineered with specific features such as pH sensitivity and modulation of surface charge, factors that enhance interaction with the cell membrane and internalization under infectious microenvironment conditions.

Thus, we can conclude that Nano_RIF is internalized by macrophages infected with Mtb, possibly due to its coating with the sulfated polysaccharide fucoidan. Studies such as those by Tukulula et al.^49^ report that rifampicin-loaded nanoparticles functionalized with specific ligands can increase intracellular drug uptake in macrophages by up to 10-fold. Thus, to studies by Cavalcanti et al.,^14^ Lira-Nogueira et al.,^12^ and Lima et al.,^42^ this polysaccharide exhibits affinity for SRA-type receptors located on the surface of macrophages. Finally, Guo et al.^39^ also suggested that other polymeric systems display similar behavior, in which nanoparticles loaded with antibiotics and modulators not only show efficient internalization but also exhibit antimicrobial synergy in cellular models, reinforcing that modulation of the delivery system may be a determining factor for therapeutic success.

## Conclusion

Studies have explored the use of different nanocarriers for anti-TB therapeutics, with most recent research focusing on drug delivery systems. The present work developed polymeric PIBCA nanoparticles coated with fucoidan and conducted in vitro biological activity assays to determine minimum and fractional inhibitory concentrations, cytotoxicity, bacterial killing kinetics, uptake kinetics, and macrophage infection, all of which yielded very promising results. Comparisons between free RIF and RIF-loaded nanoparticles found here confirm drug retention and point to evidence of antimycobacterial activity, particularly against MDR-TB strains.

This study provides evidence supporting the targeted delivery of anti-TB drugs, enabling enhanced efficacy, reduced toxicity, and consequently fewer side effects. Further studies are required to elucidate the mechanisms of intracellular uptake and trafficking pathways, as well as validation in animal models. These new technologies are expected to provide innovative solutions to combat TB and ultimately contribute to global efforts aimed at eliminating this disease.

## Data Availability

All data that support the findings of this study are included in this article. Additional raw quantifications are available from the corresponding author upon reasonable request, in accordance with institutional biosafety and regulatory requirements for handling *Mycobacterium tuberculosis* data sets, including MIC determinations, cytotoxicity assays, killing kinetics, and intracellular CFU.

## Abbreviations

Mtb: *Mycobacterium tuberculosis*
RIF: rifampicin
MIC: minimum inhibitory concentration
MDR-TB: multidrug-resistant tuberculosis
PDI: polydispersity index
CFU: colony-forming unit
DLS: dynamic light scattering

## References

[1] Bo H., Moure U. A. E., Yang Y., Pan J., Li L., Wang M., Ke X., Cui H. (2023). Mycobacterium
tuberculosis–macrophage
interaction: Molecular updates. Front. Cell.
Infect. Microbiol..

[2] Rahlwes K. C., Dias B. R. S., Campos P. C., Alvarez-Arguedas S., Shiloh M. U. (2023). Pathogenicity and virulence of Mycobacterium
tuberculosis. Virulence.

[3] World Health Organization. Global Tuberculosis Report 2024; WHO: Geneva, Switzerland, 2024. https://www.who.int/teams/global-programme-on-tuberculosis-and-lung-health/tb-reports/global-tuberculosis-report-2024. accessed March 2025.

[4] Emane A. K.
A., Guo X., Takiff H. E., Liu S. (2021). Drug resistance, fitness
and compensatory mutations in Mycobacterium tuberculosis. Tuberculosis.

[5] Goossens S. N., Sampson S. L., Van Rie A. (2020). Mechanisms
of drug-induced tolerance
in Mycobacterium tuberculosis. Clin. Microbiol.
Rev..

[6] Duarte R., Carvalho A., Ferreira D., Saleiro S., Lima R., Mota M., Raymundo E., Villar M., Correia A. (2010). Tuberculosis
treatment and management of some problems related to the medication. Rev. Port. Pneumol..

[7] Behnke M., Klemm P., Dahlke P., Shkodra B., Beringer-Siemers B., Czaplewska J. A., Stumpf S., Jordan P. M., Schubert S., Hoeppener S., Vollrath A., Werz O., Schubert U. S. (2023). Ethoxy
acetalated dextran nanoparticles for drug delivery: A comparative
study of formulation methods. Int. J. Pharm.
X.

[8] Prabhu P., Fernandes T., Chaubey P., Kaur P., Narayanan S., Ramya V. K., Sawarkar S. P. (2021). Mannose-conjugated chitosan nanoparticles
for delivery of rifampicin to osteoarticular tuberculosis. Drug Delivery Transl. Res..

[9] Beach M. A., Nayanathara U., Gao Y., Zhang C., Xiong Y., Wang Y., Such G. K. (2024). Polymeric nanoparticles
for drug
delivery. Chem. Rev.

[10] Carreiró F., Oliveira A. M., Pires A., Neves B., Nagasamy
Venkatesh D., Souto A., Durazzo E. B., Lucarini M., Eder P., Silva A. M., Santini A. (2020). Polymeric nanoparticles:
production, characterization, toxicology and ecotoxicology. Molecules.

[11] Kumar M., Virmani T., Kumar G., Deshmukh R., Sharma A., Duarte S., Brandão P., Fonte P. (2023). Nanocarriers in tuberculosis
treatment: challenges and delivery strategies. Pharmaceuticals.

[12] Lira-Nogueira M. C. B., Gibson V. P., Nicolas V., Santos-Magalhães N. S., Vauthier C. (2022). Defining endocytic pathways of fucoidan-coated PIBCA
nanoparticles from the design of their surface architecture. Pharm. Res..

[13] Howard N. C., Khader S. A. (2020). Immunometabolism
during Mycobacterium tuberculosis
infection. Trends Microbiol..

[14] Cavalcanti I. D. L., Soares J. C. S., de
Almeida S. M. V., Pessoa O., Silva E., da Silva M. S., Junior F. H. X., Manaia E. B., Ponchel G., Magalhães N. S. S. (2025). Exploring protein corona
formation on fucoidan-coated poly­(isobutyl cyanoacrylate) nanoparticles:
Implications for drug delivery. ACS Appl. Nano
Mater..

[15] Swamy N., Basavalah K., Vamsikrishna P. (2018). Stability-indicating UV-spectrophotometric
assay of rifampycin. Insight Pharm. Sci..

[16] Palomino J.-C., Martin A., Camacho M., Guerra H., Swings J., Portaels F. (2002). Resazurin microtiter
assay plate: Simple and inexpensive
method for detection of drug resistance in Mycobacterium tuberculosis. Antimicrob. Agents Chemother..

[17] Mosmann T. (1983). Rapid colorimetric
assay for cellular growth and survival: application to proliferation
and cytotoxicity assays. J. Immunol. Methods.

[18] Antibiotics in Laboratory Medicine; 5th ed., Lorian, V. ed.; Lippincott Williams & Wilkins: Philadelphia, PA, 2005.

[19] Dos
Santos A. C. D., de Souza Marinho V. H., de Aviz Silva P. H., de Matos Macchi B., Pinheiro Arruda M. S., da Silva E. O., Do Nascimento J. L. M., de Sena C. B. C. (2019). Microenvironment of Mycobacterium
smegmatis culture to induce cholesterol consumption does cell wall
remodeling and enables the formation of granuloma-like structures. Biomed. Res. Int..

[20] Zandanel C., Ponchel G., Noiray M., Vauthier C. (2021). Nanoparticles facing
the gut barrier: retention or mucosal absorption? Mechanisms and dependency
to nanoparticle characteristics. Int. J. Pharm..

[21] Mogoşanu G. D., Grumezescu A. M., Bejenaru C., Bejenaru L. E. (2016). Polymeric protective
agents for nanoparticles in drug delivery and targeting. Int. J. Pharm..

[22] Hamouda H. I., Li T., Shabana S., Hashem A. H., Yin H. (2025). Advances in fucoidan
and fucoidan oligosaccharides: Current status, future prospects, and
biological applications. Carbohydr. Polym..

[23] Stanley S., Liu Q., Fortune S. M. (2022). Mycobacterium
tuberculosis functional genetic diversity,
altered drug sensitivity, and precision medicine. Front. Cell. Infect. Microbiol..

[24] Daffé M., Marrakchi H. (2019). Unraveling the structure of the mycobacterial
envelope. Microbiol. Spectrum..

[25] Drysdale R., McEntyre J., Durinx C., Lanfear J., Blomberg N. (2020). The Annual
Indicator Monitoring and Periodic Review Processes: ELIXIR Core Data
Resources and Deposition Databases. F1000res..

[26] Xu G., Liu H., Jia X., Wang X., Xu P. (2021). Mechanisms and detection
methods of Mycobacterium tuberculosis rifampicin resistance: The phenomenon
of drug resistance is complex. Tuberculosis.

[27] Ali H. R., Ali M. R. K., Wu Y., Selim S. A., Abdelaal H. F., Nasr E. A., El-Sayed M. A. (2016). Gold nanorods
as drug delivery vehicles
for rifampicin greatly improve the efficacy of combating Mycobacterium
tuberculosis with good biocompatibility with the host cells. Bioconjugate Chem..

[28] Khatua S., Simal-Gandara J., Acharya K. (2022). Understanding immune-modulatory efficacy
in vitro. Chem. Biol. Interact..

[29] Gudgeon J., Dannoura A., Chatterjee R., Sidgwick F., Raymond B. B. A., Frey A. M., Marin-Rubio J. L., Tros M. (2025). Mass spectrometry–based
proteomic exploration of diverse murine macrophage cellular models. Life Sci. Alliance.

[30] de
O Lemos A. S., Campos L. M., de F Souza T., de L Paula P. L., da T Granato J., da Silva J. V. G., Aragão D. M. O., Rocha V. N., Coimbra E. S., Fabri R. L. (2022). Pharmacological
investigation of antioxidant and anti-inflammatory activities of aqueous
extract from Mitracarpus frigidus (Rubiaceae). J. Pharm. Pharmacol..

[31] Singh D., Malhotra P., Agarwal P., Kumar R. (2024). N-Acetyl-L-tryptophan
(NAT) ameliorates radiation-induced cell death in murine macrophages
J774A.1 via regulating redox homeostasis and mitochondrial dysfunction. J. Biochem. Mol. Toxicol..

[32] Rangaraj S., Agarwal A., Banerjee S. (2025). Bird’s
eye view on Mycobacterium
tuberculosis–HIV coinfection: understanding the molecular synergism. J. Mol. Med..

[33] Khoshnood S., Taki E., Sadeghifard N., Hassan Kaviar V., Haddadi M. H., Farshadzadeh Z., Kouhsari E., Goudarzi M., Heidary M. (2021). Mechanism of action,
resistance, synergism, and clinical
implications of delamanid against multidrug-resistant Mycobacterium
tuberculosis. Front. Microbiol..

[34] Jubilee R., Komala M., Patel S. (2024). Therapeutic potential
of resveratrol
and lignans in the management of tuberculosis. Cell Biochem. Biophys..

[35] Ishak A., Mazonakis N., Spernovasilis N., Akinosoglou K., Tsioutis C. (2025). Bactericidal versus
bacteriostatic antibacterials:
clinical significance, differences and synergistic potential in clinical
practice. J. Antimicrob. Chemother..

[36] Kim H. W., Lee J. W., Yu A.-R., Yoon H. S., Kang M., Lee B. S., Park H.-W., Lee S. K., Whang J., Kim J.-S. (2024). Isoegomaketone exhibits
potential as a new Mycobacterium
abscessus inhibitor. Front. Microbiol..

[37] Linhares L. A., dos Santos Peixoto A., de Sousa L. D. A. C., Laet J. P. L., da
Silva Santos A. C., Pereira V. R. A., Neves M. M. C., Ferreira L. F. G. R., Hernandes M. Z., de la Vega J. (2023). In vitro bioevaluation
and docking study of dihydrosphingosine and ethambutol analogues against
sensitive and multidrug-resistant Mycobacterium tuberculosis. Eur. J. Med. Chem..

[38] Birk S. E., Nielsen L. H., Boisen A. (2021). Polymeric
nano- and microparticulate
drug delivery systems for treatment of biofilms. Adv. Drug Delivery Rev..

[39] Guo Q., Guo H., Lan T., Chen Y., Chen X., Feng Y., Luo Y., Yao Y., Li Y., Pan X., Xu Y., Tao L., Liu Y., Shen X. (2021). Co-delivery
of antibiotic and baicalein
by polymeric nanoparticles with enhanced synergistic antibacterial
activity. Int. J. Pharm..

[40] Zhang, Y. ; Vilchèze, C. ; Jacobs, W. R., Jr Molecular mechanisms of drug resistance in Mycobacterium tuberculosis Tuberculosis and the Tubercle Bacillus Wiley 2004 311 151456 10.1128/9781555817657.ch8

[41] Lima
Salviano T., Dos Santos Macedo D.
C., de Siqueira
Ferraz Carvalho R., Pereira M. A., de Arruda Barbosa V. S., Dos Santos Aguiar J., Souto F. O., da Paz Carvalho da Silva M., Montenegro Pimentel L. M. L., de Ângelis Correia
de Sousa L. (2021). Fucoidan-coated liposomes: a target system to deliver
the antimicrobial drug usnic acid to macrophages infected with Mycobacterium
tuberculosis. J. Biomed. Nanotechnol..

[42] Cavalcanti I. D. L., Ximenes R. M., Pessoa O. D. L., Santos Magalhães N. S., Lira-Nogueira M. C. B. (2021). Fucoidan-coated
PIBCA nanoparticles containing oncocalyxone
A: Activity against metastatic breast cancer cells. J. Drug Delivery Sci. Technol..

[43] Bourguignon T., Godinez-Leon J. A., Gref R. (2023). Nanosized Drug Delivery Systems to
Fight Tuberculosis. Pharmaceutics.

[44] Scolari I. R., De La Cruz-Thea B., Musri M. M., Granero G. E. (2024). Quantification of
rifampicin loaded into inhaled polymeric nanoparticles by reversed
phase high-performance liquid chromatography in pulmonary nonphagocytic
cellular uptake. Anal. Methods.

[45] Rudolph J., Völkl M., Jérôme V., Scheibel T., Freitag R. (2021). Noxic effects of polystyrene
microparticles on murine
macrophages and epithelial cells. Sci. Rep..

[46] Feito M. J., Casarrubios L., Oñaderra M., Gómez-Duro M., Arribas P., Polo-Montalvo A., Vallet-Regí M., Arcos D., Portolés M. T. (2018). Response
of RAW 264.7 and J774A.1
macrophages to particles and nanoparticles of a mesoporous bioactive
glass: A comparative study. Acta Biomater..

[47] Castañeda-Fernandez C. (2022). Optimization
of Rifampicin Encapsulation in PLGA Polymeric Nanoparticles. Int. J. Pharm..

[48] Yessentayeva N. A. (2024). Optimization of PLGA-Rifampicin Nanoparticles and Antimycobacterial
Activity. Polymers.

[49] Tukulula M. (2018). Functionalization of PLGA Nanoparticles with 1,3-β-Glucan Enhances
Rifampicin Uptake. Pharm. Res..

[50] Ivashchenko O., Tomila T., Ulyanchich N., Yarmola T., Uvarova I. (2014). Fourier-transform
infrared spectroscopy of antibiotic loaded Ag-free and Ag-doped hydroxyapatites. Adv. Sci. Eng. Med..

[51] Farooq U., Ahmad T., Khan A., Sarwar R., Shafiq J., Raza Y., Ahmed A., Ullah S., Ur Rehman N., Al-Harrasi A. (2019). Rifampicin
conjugated silver nanoparticles: A new arena
for development of antibiofilm potential against methicillin resistant
Staphylococcus aureus and Klebsiella pneumoniae. Int. J. Nanomed..

[52] Lutfia F. N., Isnansetyo A., Susidarti R. A., Nursid M. (2020). Chemical composition
diversity of fucoidans isolated from three tropical brown seaweeds
(Phaeophyceae) species. Biodiversitas J. Biol.
Diversity.

